# Human MicroRNAs Interacting With SARS-CoV-2 RNA Sequences: Computational Analysis and Experimental Target Validation

**DOI:** 10.3389/fgene.2021.678994

**Published:** 2021-06-07

**Authors:** Chiara Siniscalchi, Armando Di Palo, Aniello Russo, Nicoletta Potenza

**Affiliations:** Department of Environmental, Biological and Pharmaceutical Sciences and Technologies, University of Campania “Luigi Vanvitelli”, Caserta, Italy

**Keywords:** RNA virus, microRNA, COVID-19, SARS-CoV-2, anti-viral, non-coding RNA

## Abstract

Severe acute respiratory syndrome coronavirus 2 (SARS-CoV-2) is a novel RNA virus affecting humans, causing a form of acute pulmonary respiratory disorder named COVID-19, declared a pandemic by the World Health Organization. MicroRNAs (miRNA) play an emerging and important role in the interplay between viruses and host cells. Although the impact of host miRNAs on SARS-CoV-2 infection has been predicted, experimental data are still missing. This study started by a bioinformatics prediction of cellular miRNAs potentially targeting viral RNAs; then, a number of criteria also based on experimental evidence and virus biology were applied, giving rise to eight promising binding miRNAs. Their interaction with viral sequences was experimentally validated by transfecting luciferase-based reporter plasmids carrying viral target sequences or their inverted sequences into the lung A549 cell line. Transfection of the reporter plasmids resulted in a reduction of luciferase activity for five out of the eight potential binding sites, suggesting responsiveness to endogenously expressed miRNAs. Co-transfection of the reporter plasmids along with miRNA mimics led to a further and strong reduction of luciferase activity, validating the interaction between miR-219a-2-3p, miR-30c-5p, miR-378d, miR-29a-3p, miR-15b-5p, and viral sequences. miR-15b was also able to repress plasmid-driven Spike expression. Intriguingly, the viral target sequences are fully conserved in more recent variants such as United Kingdom variant B.1.1.7 and South Africa 501Y.V2. Overall, this study provides a first experimental evidence of the interaction between specific cellular miRNAs and SARS-CoV-2 sequences, thus contributing to understanding the molecular mechanisms underlying virus infection and pathogenesis to envisage innovative therapeutic interventions and diagnostic tools.

## Introduction

Severe acute respiratory syndrome coronavirus 2 (SARS-CoV-2) is an emerging RNA virus belonging to the Betacoronavirus family, first discovered in Wuhan, China, in December 2019 (Wu et al., 2020; Zhu et al., 2020). It is an exceptionally infectious virus that has rapidly spread across the world causing a form of acute pulmonary respiratory disorder named COVID-19 (coronavirus disease 2019) that was declared a pandemic by the World Health Organization (WHO) on March 11, 2020. SARS-CoV-2 infection leads to a wide variety of clinical syndromes primarily involving the respiratory tract, ranging from asymptomatic infection to acute respiratory distress syndrome and death (approximately 3.7% mortality rate, WHO 2020). Since the declaration of the pandemic, which severely affected people health worldwide and caused global economic recession, there has been an enormous effort to investigate the virus biology in order to develop treatments and vaccines.

COVID-19 is transmitted when droplets from an infected person penetrate the respiratory tract or mucous membrane of the eyes, nose, or mouth of another person. The preferential tropism of the virus for the airways and lung epithelial cells resides in the mechanism of virus entry into the host cell: the virus uses its viral spike glycoprotein (S protein) to bind to host-cell angiotensin-converting enzyme 2 (ACE2), after which host type II transmembrane serine protease TMPRSS2 cleaves the S protein to facilitate membrane fusion; co-expression of both ACE2 and TMPRSS2 occurs in only a minority of cells, primarily secretory epithelial cells in the upper and middle respiratory tract (Hoffmann et al., 2020; Sungnak et al., 2020; Ziegler et al., 2020).

Inside the host cell, it is well recognized that viruses employ a large array of cellular components to assist genome replication, protein synthesis, and viral particle assembly. These include endoplasmic reticulum, ribosomes, tRNAs, and other cytosolic factors but also regulatory molecules such as transcription factors and microRNAs (miRNA). Canonical gene expression regulation by miRNAs is based on their binding to complementary sequences on RNA targets, requiring a perfect match with the 2–8 nucleotides of the miRNA 5′ region (seed region); then, miRNAs mediate translational repression and/or degradation of the RNA targets (Rzeszutek and Singh, 2020). As posttranscriptional regulators, miRNAs play an important role in a broad array of cellular functions, including the complex molecular interplay that is established upon viral infection of host cells (Umbach and Cullen, 2009; Trobaugh and Klimstra, 2017). Cellular miRNAs acting on viral RNAs have in fact been identified for human immunodeficiency virus (HIV) (Ahluwalia et al., 2008; Nathans et al., 2009), hepatitis C virus (Murakami et al., 2009; Li et al., 2016), hepatitis B virus (HBV) (Zhang et al., 2010; Potenza et al., 2011), papillomavirus (Nuovo et al., 2010), and many other viruses (Russo and Potenza, 2011; Trobaugh and Klimstra, 2017), including RNA viruses causing respiratory pathology such as influenza virus H1N1 and Rhino virus (Bhattacharyya and Biswas, 2020). In the case of HIV and HBV, there is convincing evidence that the identified miRNAs have been co-opted by the virus to fine-tune its replication, keeping it low to escape the immune system and establish a persistent infection (Huang et al., 2007; Mosca et al., 2014; Sagnelli et al., 2018).

To date, no experimental data have been published about microRNAs targeting SARS-CoV-2 RNA genome. However, some reports have predicted the potential of miRNA studies in the virus infection (Arisan et al., 2020; Chow and Salmena., 2020; Demirci and Adan, 2020; Guterres et al., 2020; Hosseini Rad Sm and McLellan, 2020; Jafarinejad-Farsangi et al., 2020; Nersisyan et al., 2020; Pierce et al., 2020). Among the computational analyses, the study of Chow and Salmena (2020) reported a list of 128 miRNAs potentially targeting the SARS-CoV-2 genome resulting by analysis with computer programs TargetScan and RNA22 (Chow and Salmena, 2020); another study used programs miRDB and RNAhybrid to identify 288 miRNAs with putative binding sites within the viral genome (Pierce et al., 2020). The miRanda program was used in another work and predicted 160 miRNAs targeting the viral genomic RNA (Jafarinejad-Farsangi et al., 2020). These studies encourage further investigation, but the extremely high number of predicted miRNAs makes difficult their experimental validation. We then undertook a new bioinformatic search with more restrictive parameters and taking into account the virus biology with the purpose of identifying a limited number of promising miRNAs to be immediately validated by experimental procedures.

## Materials and Methods

### Computational Analyses

**FIGURE 1:**
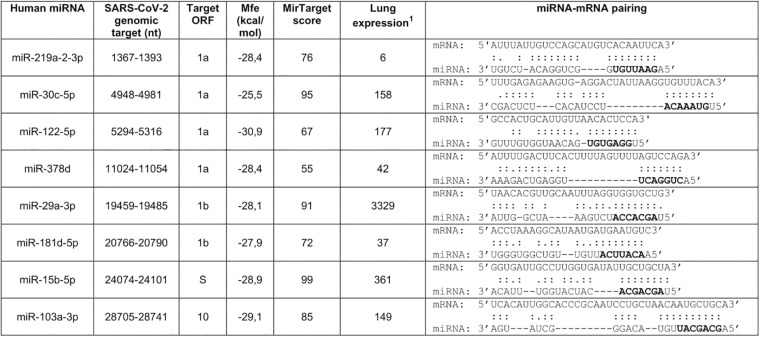
Prediction of human microRNA targets in the genomic RNA of SARS-CoV-2. Selection was performed with RNAhybrid 2.2 (Krüger and Rehmsmeier, 2006) and MirTarget (Chen and Wang, 2020). Seed regions are marked in bold. Nucleotide numbering refers to isolate Wuhan-Hu-1, complete genome (NC_045512.2). ^1^Microarrays data from the Human miRNA Tissue Atlas at https://ccb-web.cs.uni-saarland.de/tissueatlas/ (Ludwig et al., 2016). Readings of chip hybridization signals had been normalized according to Huber et al. (2002).

Prediction of microRNA target sites was performed with RNAhybrid 2.2 (Rehmsmeier et al., 2004; Krüger and Rehmsmeier, 2006), using a cutoff parameter for free energy of binding mfe < −25 kcal/mol, and MirTarget (Ivashchenko et al., 2014). The latter was used remotely within the miRDB database^1^, an online database for miRNA target prediction and functional annotations (Chen and Wang, 2020), using the predefined threshold score of 50. Conservation of the miRNA targets within the SARS-CoV-2 population was estimated by Blast searches of nucleotide collections at NCBI using as queries the entire miRNA-pairing sequences reported in Figure 1. Search was restricted to SARS-CoV-2, whose number of deposited sequences was 380 at the time of the analysis, by using its taxonomy ID (2697049). Blast-identified sequences were then downloaded and examined to verify target conservation. Nucleotide sequences of severe acute respiratory syndrome coronavirus (SARS-CoV) and Middle East respiratory-related coronavirus (MERS-CoV) genomes were downloaded from NCBI with accession numbers NC_004718.3 and NC_019843.3, respectively. Lung microRNA expression data were extracted from the Human miRNA Tissue Atlas (Ludwig et al., 2016). This is a web-based repository^2^ of experimental data collected by microarray analysis for detection of 1997 microRNAs in 61 tissues biopsies of different organs collected postmortem from two individuals.

### Reporter Constructs

The viral segments supposed to be targeted by human miRNAs were obtained by chemical synthesis of complementary oligonucleotides (Invitrogen, Thermo Fisher Scientific) containing upstream *Xho*I and *Eco*RV restriction sites and a downstream *Not*I site (Potenza et al., 2016). Each couple of oligonucleotides, representing the target sites for the different miRNAs reported in Figure 1, was annealed and ligated into *Xho*I and *Not*I sites of psiCheck-2 (Promega). *Eco*RV digestion and DNA sequencing were used to confirm the identity of the recombinant clones. A couple of oligonucleotides representing the inverted target sequences were cloned into the psiCheck-2 vector by similar approach, in order to obtain the control plasmids (indicated as I).

### Cell Cultures, Transfections, and Luciferase Assay

The human lung cell line A549 was cultured in DMEM containing 10% fetal bovine serum, 50 U/ml penicillin, and 100 μg/ml streptomycin. The day before transfection, the cells were trypsinized and seeded in a medium without antibiotics in 12-well plates.

Transfections were performed with cells at 80–90% of confluence by using 3 μl of Lipofectamine 2000 (Invitrogen, Thermo Fisher Scientific) for 1 μg of nucleic acids, as described by the manufacturer. A549 cells were transfected with 0.2 μg of reporter constructs; miScript miRNA mimics and the control unrelated sequence AllStars Negative Control (Qiagen) were transfected at 50 nM. Spike-expressing plasmid pUNO1-SARS2-S (InvivoGen) was transfected at 1 μg; after 6 h, transfection mix was replaced with complete medium. The analyses were performed 48 h after transfection.

Luciferase assays were performed using the Dual-Luciferase Reporter Assay System (Promega) according to the manufacturer’s protocol.

Experiments were independently repeated at least two times in triplicates.

Statistical comparisons were performed by Student’s *t*-test, and a value of *p* < 0.05 was considered significant.

### RNA Purification and Real-Time PCR Analysis

Total RNA purification from cell cultures was performed by miRNeasy Mini Kit (Qiagen) according to the manufacturer’s protocol. The RNA concentration was determined spectrophotometrically (NanoDrop 2000c, Thermo Fisher Scientific). RNA was retrotranscribed by SensiFAST cDNA Synthesis kit (Bioline). Then, standard SYBR Green Real-time qPCR assays were performed with the following primers: Spike, 5′-TCAACTCAGGACTTGTTCTTAC-3′ and 5′-TGGT AGGACAGGTTATCAAAC-3′ (Zhen and Berry, 2020); GAPDH (reference transcript), 5′-GAAGGTGAAGGTCGGAGTC-3′ and 5′-GAAGATGGTGATGGGATTT-3′. The expression level of Spike was normalized to the reference gene by using the 2^–ΔCt^ method.

## Results

### Prediction of miRNA Target Sites Within the SARS-CoV-2 Genome

The SARS-CoV-2 genome is made of a positive strand RNA molecule of 29903 nucleotides, provided with a cap and poly(A) tail. It contains 10 ORFs encompassing most of the genomic sequence with very short spacing regions. ORF 1a and 1b encode non-structural proteins, such as the RNA-dependent RNA Polymerase and two proteases. The other ORFs encode structural proteins including S protein and nucleocapsid polypeptide chains.

SARS-CoV-2 genomic segments potentially targeted by human microRNAs were searched by the computer programs RNAhybrid 2.2 and MirTarget which predicted 2654 and 857 pairings, respectively. Target sequences identified by both programs (about 600) were then filtered to select microRNAs expressed in the lung, the natural site of infection, and showing a perfect match of the seed region (nucleotides 2–8). Selection was based on the analysis of the first published SARS-CoV-2 genomic sequence belonging to the isolate Wuhan-Hu-1 (NC_045512.2) (Wu et al., 2020), but conservation of the target sites within the SARS-CoV-2 population was also considered. In particular, only those targets that were entirely conserved in at least 95% of the genomic sequences deposited at NCBI were selected. This procedure yielded eight highly conserved genomic sequences potentially targeted by lung microRNAs, six in ORFs 1a and 1b, one in ORF S, and one in ORF 10 (Figure 1). These data suggested that the identified miRNAs may be able to interact with the viral RNAs within infected cells, thus contributing to the regulation of SARS-CoV-2 gene expression.

### Experimental Validation

**FIGURE 2:**
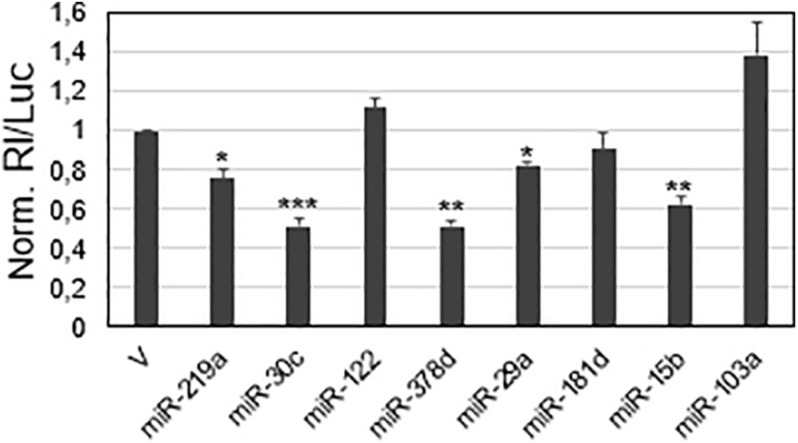
Screening of viral sequences for responsiveness to selected miRNAs in the A549 cell line. SARS-CoV-2 sequences predicted to be targeted by the indicated miRNAs were cloned in the reporter vector psiCheck-2. 48 h after transfection, luciferase activities were recorded; the Renilla luciferase activity (Rl) was normalized to the firefly luciferase activity (Luc), whose gene is also contained in the reporter vector; the values are reported as fold mean value ± s.d. relative to the luciferase activity determined for parental vector psiCheck-2 transfection (V), which was set to 1. *p*-values at Student’s *t*-test were *p < 0.05, **p < 0.01, or ***p < 0.001.

The viral sequences potentially targeted by the miRNAs were subjected to a first validation test, based on luciferase reporter constructs transfected in a cell line. A549 cells were chosen as a cell model, since they derive from the lung, the natural site of SARS-CoV-2 infection, express ACE2 and TMPRSS2 protein required for the virus entry, and are also susceptible to the virus infection (Chu et al., 2020; Pierce et al., 2020). In brief, the putative miRNA target sequences were individually cloned downstream the *Renilla reniformis* luciferase (Rl) coding sequence into the psi-Check-2 vector; the reporter constructs were then transfected in A549 cells and the luciferase activity was measured 48 h after the transfection. The expected results were that the binding ability of an endogenously expressed lung miRNA to the viral target should be registered as a reduced luciferase activity, due to the repression of the reporter protein production in comparison to the control. In this validation screening, the control was represented by the parental vector that did not contain any viral sequence. For transfection normalization, the Renilla luciferase values were related to those of luciferase (Luc), whose coding sequence is also contained in the psiCheck-2 vector. In this experimental setting, five sequences out of the eight selected by computational analyses caused luciferase activity reduction, ranging from approximately 20% inhibition (miR-219a and miR-29a target sequences) to about 40% (miR-15b target sequence) and 50% (miR-30c and miR-378d target sequences) (Figure 2).

**FIGURE 3:**
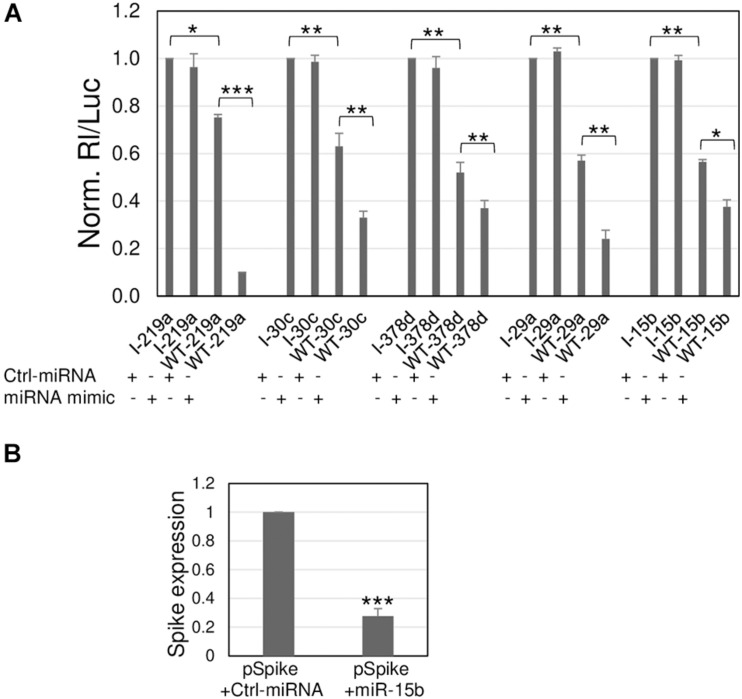
Silencing effects toward viral sequences are modulated by synthetic miRNA mimics. **(A)** A549 cells were transfected with the luciferase-based reporter constructs containing the viral target sequence of indicated miRNA (WT-miRNA abbreviation name) or the control plasmids with inverted target sequence (I-miRNA abbreviation name), along with 50 nM (+) of the specific miRNA mimic, or unrelated molecule used as negative control, Ctrl-miRNA. The values are reported as fold mean ± s.d. relative to Rl/Luc recorded for transfections of control molecules of each series (specific I construct and Ctrl-miRNA), which were set to 1. **(B)** A549 were transfected with Spike-expressing plasmid (pSpike) along with Ctrl-miRNA or miR-15b; 48 h after transfection, Spike expression was quantitated by RT-qPCR. Data are the mean ± s.d. of two independent experiments, each with three data sets. *, **, or ***, *p*-values as indicated in Figure 2.

These target sequences were subjected to further controls to exclude the possibility of an inhibition due to other factors other than the direct binding of the miRNA to the viral sequences. Control reporter constructs were prepared, each containing an inverted target sequence (I). The silencing effect of each miRNA was then investigated by transfecting the reporter construct containing the viral target sequence (now denominated WT) and its inverted sequence (I) along with a specific miRNA mimic and their negative control molecule that did not match any target sequence. The I constructs allowed to register the maximum value of the uninhibited reporter activity; in comparison, all the WT reporter plasmids transfected with the control miRNA molecule showed a reduction of luciferase activity potentially due to endogenously expressed miRNAs, thus confirming the data from the previous screening. More importantly, their co-transfection along with the miRNA mimics showed an additional and significant reduction of luciferase activity, leading to a total inhibition ranging from approximately 60% (miR-30c, miR-378d, and miR-15b) to more than 80% (miR-219a and miR-29a) in comparison to the controls (Figure 3A). Overall, the data demonstrate that the observed silencing effects can be attributed to the cellular miRNAs through a direct interaction with the viral sequences.

Given that miR-15b binds to the viral ORF encoding S protein, we further validated its binding to the S transcript by exploiting the commercially available plasmid pUNO1-SARS2-S (pSpike) expressing the natural viral S transcript from the Wuhan-Hu-1 isolate. Co-transfection of pSpike along with miR-15b mimic showed a 72% reduction of Spike expression in comparison to co-transfection along with control unrelated miRNA mimic, demonstrating the ability of miR-15b to bind the target sequence in the context of the wild-type viral transcript (Figure 3B), with the resulting silencing effect.

## Discussion

In an evolutionary perspective, viruses and humans seem to be often engaged in a zero-sum game, whereby viruses infect host cells equipped with an array of mechanisms to evade the host defense responses and replicate, and in turn host cells build a defense mechanism to restrict the infection. Cellular or viral miRNAs play an emerging and important role in that biological arms race: viruses can produce their own miRNAs that can target viral transcripts to regulate their own viral cycle or target cellular transcripts to establish a favorable host environment; on the other hand, host cells can counteract viral replication by breaking out an array of miRNAs that can directly target viral transcripts or indirectly affect virus replication by modulating host factors involved in the infection and pathogenesis (Cullen, 2006). Long-standing viral infections have probably generated a kind of equilibrium whereby viruses and humans coevolved and adapted each other also by exploiting miRNA machinery. One example is represented by HBV–human interaction: HBV infects the hepatocytes and express the transactivator HBx protein; as infection proceeds and viral load increases, the HBx level increases and leads to overexpression of miR-125a which, in turn, limits HBV replication (Mosca et al., 2014). This mechanism would balance the virus–host interaction providing a condition that is beneficial for both the host survival and the viral spread in humans; in fact, chronic hepatitis B often lasts decades. Thus, miRNA studies can contribute to understanding the mechanisms underlying the interplay between virus–host coexistence and provide a framework for the development of innovative therapeutic strategies.

Severe acute respiratory syndrome coronavirus 2 is a novel RNA virus for humans, still representing a lethal threat across the world. So far, few studies have predicted several cellular miRNAs targeting its transcripts, without experimental validation. Our research also started from a bioinformatic prediction by two different programs: RNAhybrid 2.2 and miRTarget. Then, criteria based on experimental evidence and virus preferential tropism were applied, besides the predicted binding energy and the perfect match of the seed region: confidence in the existence of the miRNA, based on number reads in miRBase (>20000) and expression in the lung, the natural site of infection. Eight targets were found in viral ORFs 1a, 1b, and S (Figure 1). Among them, miR-29a and miR-15b were also predicted to have several binding sites throughout the virus genome by Pierce et al. (2020); miR-29a emerged as a potential interactor with a viral genome also from *in silico* analysis of Jafarinejad-Farsangi et al. (2020). However, given the partial complementarity of miRNA/target pairing, the identification of the true targets is still challenging and requires experimental validation. Our experimental system consisted of luciferase-based reporter plasmids carrying viral target sequences or their inverted sequences singularly transfected or co-transfected with miRNA mimics in the lung A549 cell line. Transfection of the reporter plasmids resulted in a reduction of luciferase activity for five out of the eight potential binding sites, suggesting responsiveness to endogenously expressed miRNAs (Figure 2). Co-transfection of the reporter plasmids along with miRNA mimics led to a further and strong reduction of luciferase activity (up to 90% for miR-219a), validating the interaction between the miRNAs and viral sequences (Figure 3A). Importantly, we also demonstrated that miR-15b was able to bind to the target sequence in the context of the native viral sequence encoding S protein, strongly reducing its expression (Figure 3B).

It will be interesting to explore in specific cell contexts the cellular targets of the miRNAs identified by our analyses, in order to understand if they are also involved in the progression of SARS-CoV-2 infection and pathogenesis. So far, literature mainly reports their potential role in different types of tumors, the most studied field related to miRNA biology. In particular, miR-219a has been involved in pituitary adenomas and gastric cancer (Wang Y. et al., 2020; Yang Z. et al., 2020); miR-30c in pancreatic ductal carcinoma, laryngeal cell carcinoma, osteosarcoma, gastric and lung cancer (Cao et al., 2017; Zhou et al., 2019; Li X. et al., 2020; Tanaka et al., 2020; Yang D. et al., 2020). Intriguingly, miR-30c has also been involved in the infection by Porcine epidemic diarrhea virus (PEDV), a member of the Alphacoronavirus family: the virus engages the SOCS1/miR-30c axis to escape IFN-l-mediated antiviral immune responses (Wang C. et al., 2020). miR-29a-3p is involved in gastric and breast cancer, as well as colorectal cancer and hepatocarcinoma (Li Y. et al., 2020; Wang J. et al., 2020; Zheng et al., 2020; Pan et al., 2021; Qu et al., 2021); interestingly, miR-29a has been considered as a potential therapeutic target for HIV eradication, because of its binding to Nef viral protein, critical for viral persistence and release, and binding to the 3′UTR region of the HIV genome, mediating the transport of the virus to P-bodies (Ahluwalia et al., 2008; Nathans et al., 2009). MiR-15b is probably one of the most extensively studied in relation to cancer pathways, since it shares most of its sequence, including the seed region, with the well-known miR-15a that together with the clustered miR-16 were the first miRNAs related to a tumor, the chronic lymphocytic leukemia (Calin et al., 2002); recently, both loci, miR-15a/16-1 and miR-15b/16-2, have been shown to drive the pathogenesis of acute myeloid leukemia (Lovat et al., 2020). miR-15b has also been related to lung cancer (Wang et al., 2017; Guo et al., 2021). More importantly, two independent studies linked miR-15b to SARS-CoV-2. In the study of Kim et al. (2020), miR-15b has been predicted to bind to the genome sequences of severe acute respiratory syndrome coronavirus (SARS-CoV), Middle East respiratory-related coronavirus (MERS-CoV), and SARS-CoV-2; in addition, miR-15b was found downregulated in hamster infected lung compared to the lung samples before the infection. Consistently, Tang et al. (2020) found deregulated miR-15b expression also in red blood-depleted whole blood samples from moderate and severe COVID-19 patients and healthy donors; prediction of the miR-15b targetome and related biological pathways also supported its contribution to the disease pathogenesis, along with miR-146a-5p, miR-21-5p, and miR-142-3p, all indicated as potential biomarkers of severe COVID-19 and as candidate therapeutic targets. Besides the study of Kim et al. (2020) on miR-15b-5p, other recent computational studies take into account the conservation of the miRNA targets in other human coronavirus (Chow and Salmena, 2020; Nersisyan et al., 2020). We then verified the presence in SARS-CoV and MERS-CoV genomes of the other four experimentally validated targets and found that only the target of miR-29a-3p was present in the corresponding position SARS-CoV genome. This implies that for miR-219a-2-3p, miR-30c-5p, and miR-378d, the interactions are specific for SARS-CoV-2 whereas for miR-15b-5p and 29a-3p a wider binding ability to other coronavirus may be conceived.

Overall, those reports and our research indicate that further miRNA studies could greatly contribute to better understand the molecular mechanism underlying SARS-Co-V 2 infection and pathogenesis with relevant perspectives in diagnostics and therapeutics. Of note, our experimental data showed a strong downregulation of the reporter gene as a consequence of miRNA mimic binding to the viral sequences, up to 90% of inhibition in comparison to the control for a singularly transfected miRNA. In addition, the miR-15b mimic was able to inhibit the expression of the natural Spike transcript when it is produced by a plasmid in A549 cells.

Furthermore, the viral target sequences selected by our analyses are fully conserved in more recent variants such as United Kingdom variant B.1.1.7 and South Africa 501Y.V2.

Further research is required to evaluate if the selected miRNA mimics are able to inhibit viral translation and replication; however, our studies represent a framework to envisage innovative therapeutic interventions based on treatments able to boost the cellular reservoir of antiviral microRNAs, such as formulations of individual synthetic miRNA mimics or their cocktails as a frontline treatment for COVID-19 patients or as a preventive strategy to heighten the protective capacity of cells against the infection. Related to the last point, it would be also interesting to quantify the expression of the identified miRNAs in large cohorts of patients with different severity of the disease to understand if the potential antiviral miRNAs could have a role in the individual susceptibility to SARS-Co-V-2 infection, viral insults, and patient outcome.

## Data Availability Statement

The original contributions presented in the study are included in the article, further inquiries can be directed to the corresponding authors.

## Author Contributions

CS and AD conducted all the experiments. AR and NP conceived the study, interpreted the results, and wrote the manuscript. All authors approved the submitted version.

## Conflict of Interest

The authors declare that the research was conducted in the absence of any commercial or financial relationships that could be construed as a potential conflict of interest.

## Footnotes

1 http://mirdb.org

2 https://ccb-web.cs.uni-saarland.de/tissueatlas/
